# Supplementary material for: Pyruvate Dehydrogenase Kinase 1 inhibition mediated oxidative phosphorylation enhancement in cartilage promotes osteoarthritis progression
Source: BMC Musculoskelet Disord. 2023 Jul 20;24:597. doi: 10.1186/s12891-023-06585-6 (PMC10357736; doi:10.1186/s12891-023-06585-6)
Supplement: Supplementary file 1 — Additional file 1. [file 12891_2023_6585_MOESM1_ESM.zip › BP.Gsea.1653623667859/GOBP_REGULATION_OF_ANTIGEN_PROCESSING_AND_PRESENTATION.html]

Details for gene set GOBP\_REGULATION\_OF\_ANTIGEN\_PROCESSING\_AND\_PRESENTATION[GSEA]

|  || Dataset | 55457\_collapsed\_to\_symbols.cls.cls #OA\_versus\_normal.cls.cls #OA\_versus\_normal\_repos |
| Phenotype | cls.cls#OA\_versus\_normal\_repos |
| Upregulated in class | OA |
| GeneSet | GOBP\_REGULATION\_OF\_ANTIGEN\_PROCESSING\_AND\_PRESENTATION |
| Enrichment Score (ES) | 0.5049212 |
| Normalized Enrichment Score (NES) | 1.5610394 |
| Nominal p-value | 0.037470724 |
| FDR q-value | 0.28172922 |
| FWER p-Value | 1.0 |
Table: GSEA Results Summary

  

Fig 1: Enrichment plot: GOBP\_REGULATION\_OF\_ANTIGEN\_PROCESSING\_AND\_PRESENTATION      
 Profile of the Running ES Score & Positions of GeneSet Members on the Rank Ordered List

  

| SYMBOL | TITLE | RANK IN GENE LIST | RANK METRIC SCORE | RUNNING ES | CORE ENRICHMENT || 1 | HFE | homeostatic iron regulator [Source:HGNC Symbol;Acc:HGNC:4886] | 16 | 1.130 | 0.1897 | Yes |
| 2 | TREM2 | triggering receptor expressed on myeloid cells 2 [Source:HGNC Symbol;Acc:HGNC:17761] | 342 | 0.726 | 0.2872 | Yes |
| 3 | TAPBPL | TAP binding protein like [Source:HGNC Symbol;Acc:HGNC:30683] | 789 | 0.572 | 0.3492 | Yes |
| 4 | PYCARD | PYD and CARD domain containing [Source:HGNC Symbol;Acc:HGNC:16608] | 1164 | 0.484 | 0.4019 | Yes |
| 5 | FCGR2B | Fc fragment of IgG receptor IIb [Source:HGNC Symbol;Acc:HGNC:3618] | 2016 | 0.360 | 0.3967 | Yes |
| 6 | CCL19 | C-C motif chemokine ligand 19 [Source:HGNC Symbol;Acc:HGNC:10617] | 2060 | 0.355 | 0.4534 | Yes |
| 7 | HLA-DOB | "major histocompatibility complex, class II, DO beta [Source:HGNC Symbol;Acc:HGNC:4937]" | 2147 | 0.344 | 0.5049 | Yes |
| 8 | NOD2 | nucleotide binding oligomerization domain containing 2 [Source:HGNC Symbol;Acc:HGNC:5331] | 3154 | 0.235 | 0.4665 | No |
| 9 | YTHDF1 | YTH N6-methyladenosine RNA binding protein 1 [Source:HGNC Symbol;Acc:HGNC:15867] | 4320 | 0.127 | 0.3974 | No |
| 10 | HLA-DOA | "major histocompatibility complex, class II, DO alpha [Source:HGNC Symbol;Acc:HGNC:4936]" | 4344 | 0.125 | 0.4167 | No |
| 11 | CCR7 | C-C motif chemokine receptor 7 [Source:HGNC Symbol;Acc:HGNC:1608] | 5678 | 0.017 | 0.3161 | No |
| 12 | FGL2 | fibrinogen like 2 [Source:HGNC Symbol;Acc:HGNC:3696] | 6125 | -0.014 | 0.2839 | No |
| 13 | CD74 | CD74 molecule [Source:HGNC Symbol;Acc:HGNC:1697] | 6374 | -0.033 | 0.2703 | No |
| 14 | WAS | WASP actin nucleation promoting factor [Source:HGNC Symbol;Acc:HGNC:12731] | 6666 | -0.054 | 0.2568 | No |
| 15 | CCL21 | C-C motif chemokine ligand 21 [Source:HGNC Symbol;Acc:HGNC:10620] | 6856 | -0.068 | 0.2536 | No |
| 16 | SLC11A1 | solute carrier family 11 member 1 [Source:HGNC Symbol;Acc:HGNC:10907] | 7153 | -0.092 | 0.2462 | No |
| 17 | LILRB2 | leukocyte immunoglobulin like receptor B2 [Source:HGNC Symbol;Acc:HGNC:6606] | 9317 | -0.257 | 0.1216 | No |
| 18 | NOD1 | nucleotide binding oligomerization domain containing 1 [Source:HGNC Symbol;Acc:HGNC:16390] | 10953 | -0.424 | 0.0664 | No |
| 19 | THBS1 | thrombospondin 1 [Source:HGNC Symbol;Acc:HGNC:11785] | 11498 | -0.501 | 0.1088 | No |
Table: GSEA details [plain text format]

  

Fig 2: GOBP\_REGULATION\_OF\_ANTIGEN\_PROCESSING\_AND\_PRESENTATION      
 Blue-Pink O' Gram in the Space of the Analyzed GeneSet

  

Fig 3: GOBP\_REGULATION\_OF\_ANTIGEN\_PROCESSING\_AND\_PRESENTATION: Random ES distribution      
 Gene set null distribution of ES for **GOBP\_REGULATION\_OF\_ANTIGEN\_PROCESSING\_AND\_PRESENTATION**
